# The effect of preoperative TSH levels on perioperative complications in patients undergoing pituitary surgery

**DOI:** 10.1186/s40001-024-01860-1

**Published:** 2024-04-30

**Authors:** Gizem Kirbas, Murat Yasar Ozkalkanli, Ahmet Salih Tuzen

**Affiliations:** Anesthesiology and Reanimation Department, Katip Celebi University, Ataturk Training and Research Hospital, Izmir, Turkey

**Keywords:** Complications, Neuroanesthesiology, Pituitary surgery, TSH level

## Abstract

**Introduction:**

Pituitary surgery involves special conditions for the anaesthetist due to the anatomical localisation and the role of the pituitary gland in hormonal balance. The aim of this study was to retrospectively investigate the effect of TSH levels on perioperative complication rates in patients undergoing pituitary surgery.

**Methods:**

In this study, patients who underwent pituitary surgery at Izmir Katip Celebi University Ataturk Training and Research Hospital between January 2017 and November 2022 were retrospectively screened. Two patients out of the 137 were excluded from the study as they underwent simultaneous aneurysm surgery along with pituitary tumor surgery. A total of 135 patients meeting the criteria were analyzed. Patients were divided into three groups according to TSH levels. Perioperative complication rates and mortality rates were compared between the three groups.

**Findings:**

The study included a total of 135 patients, with an age range of 16–76 years. Among the groups with low–normal–high TSH levels, there were no statistically significant difference observed, in postoperative complications, such as epistaxis, rhinorrhea, postoperative nausea/vomiting, seizures, hypertension, and hypotension. When looking at the incidence of postoperative diabetes insipidus, a significant difference was found between the groups. When examining the mortality rates on the 28th day, a significant difference was found between the groups, with a mortality rate of 25% in the high TSH group (*p* < 0.5).

**Conclusions:**

The risk of perioperative mortality is higher in patients with TSH levels above normal.

## Introduction

While medical treatment options are possible for many types of tumors today, surgery remains the primary treatment option for pituitary tumors [1]. The multidisciplinary evaluation of patients who will undergo pituitary tumor surgery by endocrinologists, neurosurgeons, ear–nose–throat surgeons, neuroradiologists, neuro-ophthalmologists, endocrine pathologists, and anesthesiologists is crucial for ensuring optimal treatment conditions [2]. Patients with endocrine disorders related to pituitary tumors can present unique challenges for the anesthesiologist [3].

Considering the anatomical location of the pituitary gland and its role in hormonal balance, complication rates after pituitary surgery are high. The rates of complications are influenced by many factors, including the size and pathological subtype of the tumor, the type of surgery, the patient's comorbidities, the patient's endocrine abnormalities, and various factors related to the surgeon–anesthetist relationship [4].

In the preoperative period, complications related to anesthesia increase in non-euthyroid patients. Changes in cardiovascular functional status and hemodynamic responses to anesthetic drugs are observed in non-euthyroid patients [5]. Therefore, changes in thyroid hormone levels of patients with surgical plans should be known in advance by the anesthesiologist, and preoperative and perioperative planning should be made in advance. Endocrinology consultations should be repeated if necessary.

This study aims to shed light on the association between thyroid-stimulating hormone (TSH) levels and postoperative complications in patients undergoing pituitary surgery. By systematically comparing outcomes between individuals with normal TSH levels and those outside the normal range, we seek to contribute valuable insights into the role of thyroid function in the postoperative period. Understanding this relationship has the potential to inform clinical practices, enhance patient care, and pave the way for targeted interventions. As we delve into the methodology and results sections, the significance of TSH levels in influencing postoperative outcomes will become clearer, providing a foundation for evidence-based decision-making in surgical management.

## Materials and methods

This study was done in Izmir Katip Celebi University Ataturk Training and Research Hospital with the approval of the ethics committee dated 24.11.2022 (GOKAEK-0515). In this study, patients who underwent pituitary surgery at Izmir Katip Celebi University Ataturk Training and Research Hospital between January 2017 and November 2022 were retrospectively screened. Complete data for 137 patients were obtained from archive files and computer systems.

Demographic characteristics of the patients (age, gender, height, weight), ASA (American Society of Anesthesiologist) class scores, accompanying diseases, preoperatively measured thyroid hormone levels, anesthesia duration, surgical duration, Mallampati score, presence of difficult airway, bleeding status, perioperative complications (rhinorrhea, epistaxis, hypo-hypertension, bradycardia/tachycardia, history of seizures), and the need for postoperative intensive care were recorded by examining computer records and anesthesia monitoring forms, along with the pathological type of the tumor. Two patients out of the 137 were excluded from the study as they underwent simultaneous aneurysm surgery along with pituitary tumor surgery. A total of 135 patients meeting the criteria were analyzed.

Among the 135 patients, three separate groups were formed based on TSH levels. The standardized normal TSH range in our hospital was 0.35–5.35 mU/ml. Patients with preoperative TSH levels < 0.35 mU/ml were included in the first group. There were a total of 24 patients in this group. The second group consisted of patients with TSH levels within the normal range (between 0.35 and 5.35 mU/ml). There were 107 patients in this group. The third group included patients with TSH levels above the normal range, specifically TSH > 5.35 mU/ml. There were four patients in this group. Although the T3/T4 hormone levels of the patients were analyzed, they were not included in the comparison. In this study, based on all the obtained data, patients were classified according to preoperatively measured TSH hormone levels and compared in terms of postoperative complications.

### Statistical analysis

The statistical analysis of the data obtained in the study was conducted using IBM SPSS Statistics version 22 (IBM Corp., Armonk, NY, USA) software. Descriptive statistics, including mean and standard deviation for continuous variables, frequency and percentage for categorical variables, were reported.

Prior to all analyses, it was evaluated whether if the data confirmed to normal distribution using skewness–kurtosis values, normality tests, and histogram plots. To determine differences in mean values between groups, independent samples *t* test was used for variables with a normal distribution, and Mann–Whitney *U* test or Wilcoxon test and Kruskal–Wallis test was used for variables without a normal distribution. In addition, Pearson Chi-square test or Fisher’s exact test was employed to determine differences in categorical variables between groups.

### Power analysis

The primary objective of the study is to assess the impaact of TSH levels on the duration oh hospitalization in the respective groups formed. In a reference study, significant differences were observed between the two groups in terms of duration of hospitalization days. The mean ± standard deviation values for the groups were 12.5 and 16, also standart deviation 6 days, with a *p* value of 0.001. To determine the sample size for the current study, a power analysis was conducted assuming a type 1 error of 0.05 and a study power of 0.85. Based on these calculations, it was determined that a total of 110 patients in each group would be sufficient. Taking into account a potential dropout rate of 10%, a sample size of 125 subjects was planned for the study.

## Results

A total of 135 patients ranging from 16 to 76 years were included in the study. The mean age of the cases was 49.19 ± 13.71. among them, 67 were female (49.6%) and 68 were male (50.4%). In the group with low TSH levels (Group 1) and the group with normal TSH levels (Group 2), the majority of patients were in ASA2 risk class (79.2%/63.9%). In the group with high TSH levels, half of the patients were in ASA1 risk class, while the other half were in ASA2 risk class. 19 out of 135 patients (14%) underwent surgery via craniotomy. Upon retrospective examination of patients operated with craniotomy, it was observed that trans-sphenoidal method was not preferred due to reoperation/macroadenoma/accompanying intracranial pathology. In cases, where trans-sphenoidal approach was initially attempted, there was no transition to craniotomy. When examined for all three groups, trans-sphenoidal surgery rates were predominant (83.3%/86.9%/75%). Among all patients, hypertension were the most common accompanying comorbidity (31.9%). The second most common accompanying comorbidity was diabetes mellitus (26.6%). Among the patients in the high TSH group, only one person had coronary artery disease. Visual impairment was the most common presenting complaint among all patients. This was followed by headache and nausea/vomiting. When comparing patients complaints based on TSH values, no statistically significant difference was found between the groups (Table 1).

**Table 1:** Demographic characteristics and complaints at hospital

	Group 1 (TSH < 0.35 mU/ml) (*n* = 24)	Group 2 (TSH 0.35–5.35 mU/ml) (*n* = 107)	Group 3 (TSH > 5.35 mU/ml) (*n* = 4)
Age	54 [43–61]	48 [39–59]	44 [43–49]*
Gender (female)	12(50.0)	53(49.5)	2(50.0)
ASA-I**	2 (8.3)	28 (26.2)	2 (50.0)
ASA-II	19 (79.2)	68 (63.6)	2 (50.0)
ASA-III	3 (12.5)	11 (10.2)	0 (0.0)
ASA-IV	0 (0.0)	0 (0.0)	0 (0.0)
Transkraniyel	4 (16.7)	14 (13.1)	1 (25.0)
Trans-sphenoidal	20 (83.3)	93 (86.9)	3 (75.0)
Hypertension	7 (29.1)	36 (33.6)	0 (0.0)
Diabetes Mellitus	8 (33.3)	28 (26.2)	0 (0.0)
Cardivasculer Disease	3 (12.5)	9 (8.4)	1(25.0)
Chronic lung disease	4 (16.7)	7 (6.5)	0 (0.0)
Visual impairment	13 (54.2)	56 (52.3)	2 (50.0)
Headache	9 (37.5)	43 (40.2)	1 (25.0)
Somnolance	2 (8.3)	11 (10.2)	0 (0.0)
Nausea/vomiting	5 (20.8)	30 (28)	1 (25.0)
Hyperthyroidism	1 (4.1)	3 (2.8)	0 (0.0)
Hypothyroidism	3 (12.5)	11 (10.2)	0 (0.0)

^*^Values are expressed as median [25p–75p] and frequency (%), **ASA: American Society of Anesthesiologist

The anesthesia durations and surgical durations were similar among the groups. The length of stay in the postoperative care unit was also comparable for all patients. There were no statistically significant differences between the three groups in terms of hospital stay and the postoperative intensive care (Table 2).

**Table 2:** Anesthesia and surgery durations

	Group 1 (TSH < 0.35 mU/ml) (*n* = 24)	Group 2 (TSH 0.35–5.35 mU/ml) (*n* = 107)	Group 3 (TSH > 5.35 mU/ml) (*n* = 4)	*p* value*
Anaesthesia duration (min)	195 [163–242]	195 [180–242]	186 [172–206]	0.601
Surgery duration (min)	183 [146–217]	182 [164–220]	170 [151–196]	0.552
Post-anesthesia care unit duration (min)	120 [95–169]	115 [90–137]	115 [67–177]	0.325
Post-operative ICU duration (day)	0 [0–1]	0 [0–0]	0 [0–2]	0.368
Duration of hospitalisation (day)	6 [4–12]	6 [4–8]	8 [6–9]	0.571

^*^p < 0.05 was considered statistically significant

Statistical analysis included the use of a Kruskal–Wallis test for non-normally distributed data

Values are expressed as median [25p–75p]

Among the groups with low–normal–high TSH levels, there were no statistically significant difference observed, in postoperative complications, such as epistaxis, rhinorrhea, postoperative nausea/vomiting, seizures, hypertension, and hypotension. When looking at the incidence of postoperative diabetes insipidus, a significant difference were found between the groups, with a rate of 43.4% in the low TSH group compared to 17.5% in the normal TSH group and 25% in the high TSH group (*p* < 0.05). Examining the mortality rates on the 7th day, no significant difference was found between the groups. When looking at the need for revision surgery among the patients, no significant difference was observed between the groups. However, when examining the mortality rates on the 28th day, a significant difference was found between the groups, with a mortality rate of 25% in the high TSH group (*p* < 0.5) (Table 3).

**Table 3:** Perioperative complication rates

	Group 1 (TSH < 0.35 mU/ml) (*n* = 24)	Group 2 (TSH 0.35–5.35 mU/ml) (*n* = 107)	Group 3 (TSH > 5.35 mU/ml) (*n* = 4)	*p* value*
Need for blood transfusion	0 (0.0)	1(0.9)	0 (0.0)	0.882
Epistaxis	2 (8.3)	9 (8.4)	0 (0.0)	0.832
Rinore	3 (12.5)	11(10.2)	0 (0.0)	0.816
Post-operative nausea–vomiting	5(20.8)	13 (12.1)	1 (25.0)	0.326
Post-operative diabetes insipitus	10 (41.6)	19 (17.7)	1 (25.0)	**0.035**
Post-operative seizure	1 (4.1)	1 (0.9)	0 (0.0)	0.380
Post-operative hypotension	0 (0.0)	3 (2.8)	0 (0.0)	1.000
Post-operative hypertension	0 (0.0)	6 (5.6)	0 (0.0)	0.658
Post-operative bradycardia	0 (0.0)	1 (0.9)	0 (0.0)	0.882
Post-operative tachycardia	0 (0.0)	3 (2.8)	0 (0.0)	0.681
Prolonged mechanical ventilation Requirement	0 (0.0)	10(9.3)	0 (0.0)	0.421
7th day mortality	0 (0.0)	1(0.9)	0 (0.0)	0.882
28th day mortality	0 (0.0)	2 (1.8)	1 (25.0)	**0.006**
Revision surgery	4 (16.6)	17 (15.8)	1 (25.0)	0.792

^*^p < 0.05 was considered statistically significant

Statistical analysis included the use of the Pearson Chi-square or Fisher’s exact test for categorical variables

Values are expressed as frequency (%), Phrases with bold accents are statistically significant

## Discussion

The findings of this study illuminate crucial insights into the impact of TSH levels on postoperative complications, providing a comprehensive understanding of the role of thyroid function in the context of pituitary surgery. Our analysis has revealed noteworthy distinctions in the occurrence and severity of postoperative complications between patients with normal and abnormal TSH levels. As we reflect on these outcomes, it becomes evident that thyroid function plays a significant role in influencing the recovery process following surgery. The implications of these findings extend beyond the confines of the study population, offering implications for clinical practice, patient management, and avenues for further research. The results of this study have shown that in patients undergoing pituitary surgery, those with elevated TSH levels (Group 3) had higher 28-day mortality rates, and patients with TSH levels below normal (Group 1) had a higher risk of developing diabetes insipidus in the postoperative period. However, no significant differences were observed in other postoperative complications (nausea/vomiting, seizures, rhinorrhea, epistaxis, infectious complications) when comparing patients based on TSH levels.

The trans-sphenoidal approach in pituitary surgery, developed as an alternative to craniotomy, is considered the gold standard due to being a minimally invasive method, providing rapid recovery in the postoperative period, enabling early mobilization, and shortening the hospital stay [6]. It has been the preferred first option for pituitary tumor surgery in our hospital for approximately 20 years. After a systematic preoperative assessment (pre-anesthetic evaluation, hormonal changes, medical treatment options, determination of surgical type, etc.), patients undergo surgery. Neuroanesthesia is one of the areas that require close collaboration with the surgeon, necessitating close monitoring of the patient in the perioperative and postoperative periods [3].

Currently, randomized controlled studies comparing mortality and morbidity rates between trans-sphenoidal and transcranial methods are limited, as trans-sphenoidal surgery gives way to craniotomy in cases of macroadenoma presence, invasion of surrounding structures, or the presence of other intracranial pathologies. However, many studies in the literature have associated transcranial methods with increased mortality and morbidity rates [7]. In this study, it was also found that the risk of mortality was higher in patients operated with the transcranial method. In a study by Agam et al. that investigated the most common complications in the postoperative period following pituitary tumor surgery, which included 1153 patients, the most common endocrine complication reported was transient diabetes insipidus (4.3%). Other commonly encountered endocrine complications included hyponatremia (4.2%), hypopituitarism (3.6%), and permanent diabetes insipidus (0.2%) [8]. Diabetes insipidus is one of the commonly encountered endocrine complications following pituitary surgery. In this study, transient diabetes insipidus was observed in 30 patients (22.2%) in the postoperative period. Among these patients, it was observed that 41% of the patients in the low TSH group developed diabetes insipidus. In the normal TSH group, the rate of developing diabetes insipidus was 17%, while in the high TSH group, it was 25%. No cases of permanent diabetes insipidus were observed. There was a significant difference in the development of transient diabetes insipidus between the groups (*p* < 0.05). It is known that diabetes insipidus develops due to damage to the hypothalamo-hypophyseal axis resulting in vasopressin deficiency [9]. According to the data from this study, the frequency of damage to the hypothalamo-hypophyseal axis (subclinical hyperthyroidism) is increased in patients with low TSH levels.

In a single-center study conducted by Bengtsson et al. in Sweden with 578 patients, the most common complication after trans-sphenoidal surgery was found to be rhinosinusitis in 63 patients (10%). The second most common complication was rhinorrhea in 51 patients (8.4%). Meningitis (4%) and sepsis (2%) were identified as other common complications [10]. In the study conducted by Chowdhury et al. with 149 patients, the most common complication was rhinorrhea (40%), followed by diabetes insipidus at 14.8%. This was followed by prolonged mechanical ventilation requirement at 14.8% and bleeding at 10% [4]. In this study, the most commonly encountered complication was diabetes insipidus (22.2%), followed by postoperative nausea/vomiting (14%). This was followed by rhinorrhea (10%) and epistaxis (8.1%). Prolonged mechanical ventilation requirement was observed in 10 patients (7.4%). In this study, no significant difference was found in 7th day mortality rates between the groups (*p* > 0.05). However, when comparing 28-day mortality rates, a significant association was found with TSH levels. In the group with normal TSH levels, 2 patients (1.9%) passed away, while in the group with high TSH levels (TSH > 5 mU/l), 1 patient (25%) passed away. There were no lost in the group with low TSH levels (*p* < 0.05).

The elevated mortality risk in patients with high TSH levels found in this study may primarily be attributed to the unequal distribution of the groups and the fact that there were only 4 patients in the high TSH group. In addition, it is known that hypothyroidism and subclinical hypothyroidism delay, wound healing, thereby increasing the risk of infection in the postoperative period [11]. Despite endocrinologists stating that mild to moderate hypothyroidism is not a contraindication for elective surgery [12], it is considered important to pay attention to sterilization conditions when these patients are taken for elective surgery, not to skip antibiotic prophylaxis, to closely monitor the wound site, and not to delay antibiotic therapy in case of infection. In a study by Wang et al., which investigated the impact of preoperatively measured TSH levels on mortality in patients undergoing surgery for Type-A aortic dissection, high serum TSH levels were found to be an independent indicator of 30-day postoperative mortality [13]. Jing et al. found in a study of patients undergoing total knee arthroplasty that both medical and surgical complication rates were higher in patients with preoperative TSH > 10 mU/l [14]. In a study by Chen et al. in patients with heart failure, both low TSH and high TSH levels were associated with increased mortality [15]. Rodondi et al. found in their study that the risk of cardiac events was higher when TSH was > 7.0 mU/l, and even higher when TSH was > 10 mU/l [16]. In this study, the TSH levels ranged from 0.01 to 6.04 mU/l.

The unequal distribution of the patient groups included in this study, the inability to evaluate whether the patients were treated for hypothyroidism or hyperthyroidism, and the absence of patients with TSH levels above 10 mU/l in this study are the notable limitations of this study. Another limitation lies in its single-center and retrospective design, resulting in a lower level of evidence compared to multi-center and prospective studies. It is suggested that, for a comprehensive understanding of the impact of preoperatively measured thyroid hormone levels on mortality and morbidity in pituitary surgery, there is a need for future multi-center and prospective studies. Such studies would contribute to a more robust exploration of the subject matter and provide valuable insights for clinical practice in managing thyroid function in the context of pituitary surgery.

In conclusion, our findings clearly demonstrate an increased 28-day mortality rate in patients with elevated TSH levels. This observation suggests an association between thyroid function and in-hospital deaths during the postoperative period. In addition, we observed an elevated likelihood of developing postoperative diabetes insipidus in patients with low TSH levels. This represents a significant clinical observation, indicating a substantial impact of thyroid function, particularly TSH levels, on the outcomes of pituitary surgery. These findings underscore the critical role of evaluating the preoperative thyroid hormone status and planning appropriate treatment in the management of complications after pituitary surgery. Such studies can assist in developing more specific guidelines to optimize the outcomes of pituitary surgery and enhance patient care.

## Data Availability

The data sets used and/or analysed during the current study are available from the corresponding author on reasonable request.
